# Fe-S cofactors in the SARS-CoV-2 RNA-dependent RNA polymerase are potential antiviral targets

**DOI:** 10.1126/science.abi5224

**Published:** 2021-06-03

**Authors:** Nunziata Maio, Bernard A. P. Lafont, Debangsu Sil, Yan Li, J. Martin Bollinger, Carsten Krebs, Theodore C. Pierson, W. Marston Linehan, Tracey A. Rouault

**Affiliations:** 1 Eunice Kennedy Shriver National Institute of Child Health and Human Development, National Institutes of Health, Bethesda, MD 20892, USA.; 2 SARS-CoV-2 Virology Core, Laboratory of Viral Diseases, Division of Intramural Research, National Institute of Allergy and Infectious Diseases, National Institutes of Health, Bethesda, MD 20892, USA.; 3 Department of Chemistry, The Pennsylvania State University, University Park, PA 16802, USA.; 4 Proteomics Core Facility, National Institute of Neurological Disorders and Stroke, National Institutes of Health, Bethesda, MD 20892, USA.; 5 Department of Biochemistry and Molecular Biology, The Pennsylvania State University, University Park, PA 16802, USA.; 6 Laboratory of Viral Diseases, Division of Intramural Research, National Institute of Allergy and Infectious Diseases, National Institutes of Health, Bethesda, MD 20892, USA.; 7 Urologic Oncology Branch, Center for Cancer Research, National Cancer Institute, Bethesda, MD 20892, USA.

## Abstract

Severe acute respiratory syndrome coronavirus 2 (SARS-CoV-2), the causal agent of COVID-19, uses an RNA-dependent RNA polymerase (RdRp) for the replication of its genome and the transcription of its genes. We found that the catalytic subunit of the RdRp, nsp12, ligates two iron-sulfur metal cofactors in sites that were modeled as zinc centers in the available cryo–electron microscopy structures of the RdRp complex. These metal binding sites are essential for replication and for interaction with the viral helicase. Oxidation of the clusters by the stable nitroxide TEMPOL caused their disassembly, potently inhibited the RdRp, and blocked SARS-CoV-2 replication in cell culture. These iron-sulfur clusters thus serve as cofactors for the SARS-CoV-2 RdRp and are targets for therapy of COVID-19.

## Mind your metals

Iron–sulfur clusters are important cofactors for proteins involved in metabolism and electron transfer but are also sometimes found in enzymes involved in transcription and replication of DNA. In vitro expression of such enzymes can result in faulty cluster assembly and confusion about the composition of the functional enzyme. Using a careful anoxic purification scheme, Maio *et al.* found that the severe acute respiratory syndrome coronavirus 2 RNA–dependent RNA polymerase contains two iron–sulfur clusters at two sites previously observed to bind zinc ions. Mutation of the ligating cysteine residues resulted in loss of polymerase activity. A less severe loss of activity was seen in the zinc-containing enzyme. Treatment with the nitroxide drug TEMPOL resulted in degradation of the clusters, enzyme inhibition, and inhibition of viral replication in cell culture.

*Science*, abi5224, this issue p. 236

## Teaser

Targeting RNA-dependent RNA polymerase Fe-S clusters inhibits SARS-CoV-2 infection in vitro.

The novel coronavirus severe acute respiratory syndrome coronavirus 2 (SARS-CoV-2) has caused a global pandemic known as COVID-19 (*1*–*3*), which can be prevented by vaccines but for which antiviral treatments are much needed. Coronaviruses employ a multisubunit machinery for replication and transcription. A set of nonstructural proteins (nsps) produced as cleavage products of the ORF1a and ORF1ab polyproteins (*4*) assemble to facilitate viral replication and transcription. The core component of this complex is the catalytic subunit (nsp12) of an RNA-dependent RNA polymerase (RdRp) (*5*), which catalyzes the synthesis of viral RNA and thus plays a central role in the replication and transcription cycle of SARS-CoV-2, with the assistance of nsp7 and nsp8 as accessory factors (*6*, *7*). Structures of the RdRp (nsp12-nsp7-nsp8 complex) alone and in complex with the helicase have been determined by cryo–electron microscopy (cryo-EM) (*8*–*11*); in all of these structures, the RdRp of SARS-CoV-2 was proposed to contain zinc ions ligated in the same locations as those observed in SARS-CoV (*7*) in highly conserved metal binding motifs composed of H295-C301-C306-C310 and C487-H642-C645-C646 (fig. S1). These zinc ions have been proposed to serve a structural role in maintaining the integrity of the RdRp architecture (*7*–*11*) (see supplementary text in the supplementary materials). Zinc has long been known to be capable of replacing endogenous iron-sulfur (Fe-S) metal cofactors during standard aerobic purification of proteins (*12*–*15*), because Fe-S clusters are inherently susceptible to destabilization and degradation by oxidants, including oxygen, superoxide (O_2_^−^), and nitric oxide (*16*). Notably, Fe-S clusters, inorganic cofactors often associated with biological redox reactions (*17*, *18*), have been identified in numerous proteins involved in DNA and RNA metabolism, where they play a variety of critical functional roles (*12*, *13*, *19*–*26*).

**Fig. 1:**
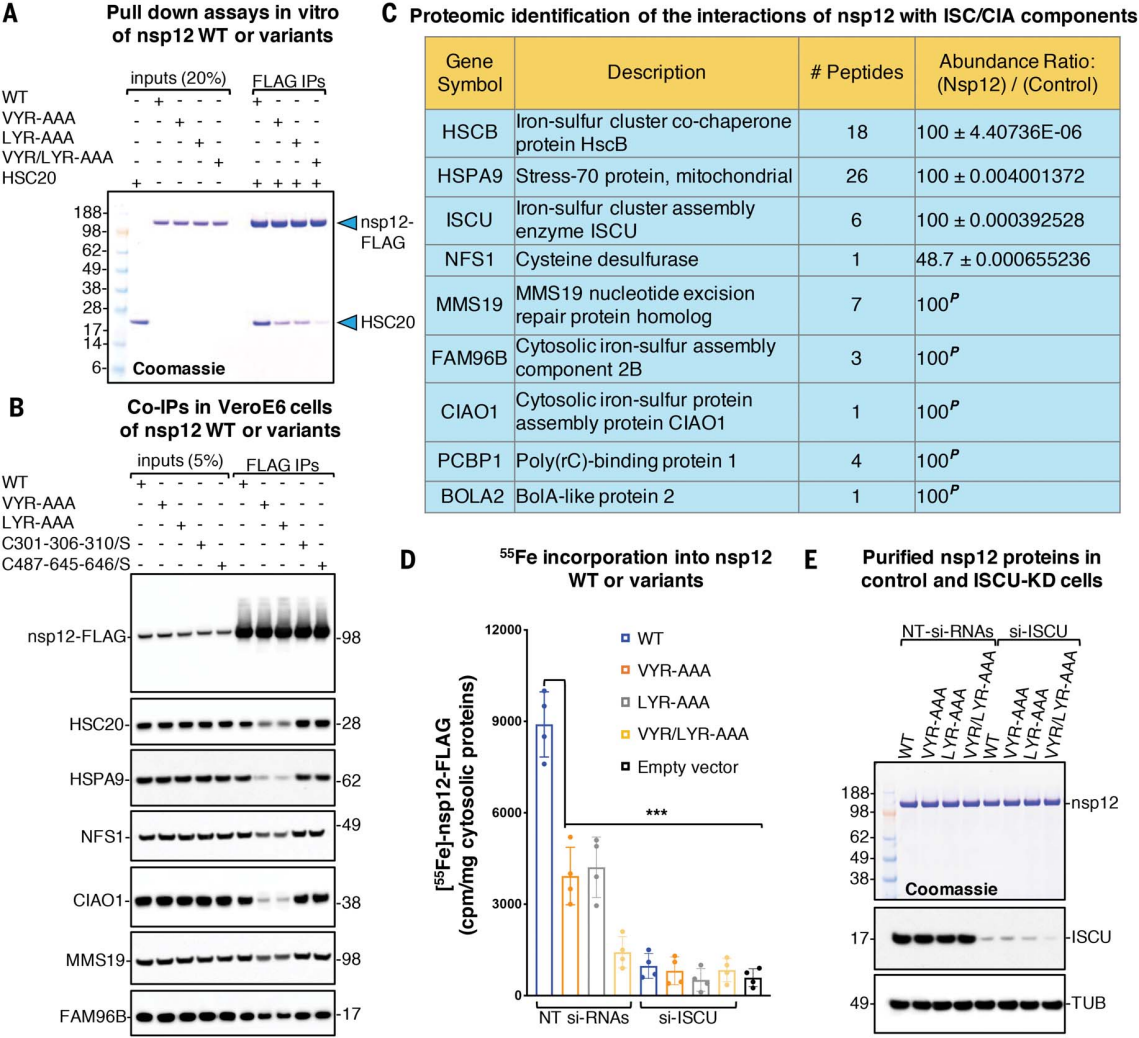
Fe-S cluster incorporation into nsp12 occurs through its interactions with components of the Fe-S biogenesis machinery. (**A**) Representative Coomassie blue staining of pull-down assays performed with purified proteins. Purified nsp12-FLAG (0.25 μg) or the variants wherein either or both LYR motifs were replaced by alanines (VYR-AAA, LYR-AAA, and VYR/LYR-AAA, respectively) were combined with 0.25 μg of HSC20, as indicated. Immunoprecipitations (IPs) were performed with anti-FLAG antibody to immunocapture nsp12 proteins. The presence of HSC20 (i.e., HSCB) in the eluates after IPs of nsp12 proteins was analyzed by SDS–polyacrylamide gel electrophoresis and Coomassie staining. Aliquots corresponding to 20% of the inputs were run on the gel for comparison (*n* = 5 biological replicates). (**B**) Eluates after IPs of nsp12 WT or variants recombinantly expressed in Vero E6 cells, as indicated, were probed with antibodies against FLAG to verify the efficiency of IP and against components of the Fe-S cluster (HSC20, HSPA9, and NFS1) and of the cytoplasmic Fe-S (CIA) assembly machinery (CIAO1, MMS19, and FAM96B) (*n* = 6). (**C**) Mass spectrometry identification of affinity purified interacting partners of nsp12 that are components of the Fe-S cluster biogenesis pathway (see data S1 for a complete list). The protein ratios were calculated as reported in the methods (*n* = 6). The maximum allowed fold change value was set to 100. In the instances (marked with a superscript *P*) in which the interacting partner was detected in the nsp12-only samples and not in the negative controls, the nsp12/control ratios were set to 100 and reported without *P* values. (**D**) Levels of radiolabeled iron (^55^Fe) incorporated into nsp12 WT or the variants in control cells transfected with nontargeting siRNAs (NT siRNAs) and in cells transfected with siRNAs directed against the main scaffold protein ISCU (si-ISCU). Levels of iron stochastically associated with the beads in lysates from cells transfected with the backbone plasmid (empty-vector, p3XFLAG-CMV-14) are also reported (accounting for 587 ± 292.62 cpm/mg of cytosolic proteins) and were not subtracted from measurements of radiolabeled iron incorporated into nsp12 WT or variants in the chart (*n* = 4). Significance was determined by two-way analysis of variance (ANOVA) and Sidak’s multiple comparisons test. Mean ± 95% confidence interval (CI). ***P < 0.001. (**E**) Representative Coomassie staining showing levels of nsp12 WT or variants in control and ISCU-depleted cells that were quantified in (D) for their iron content. Immunoblots to ISCU, showing the efficiency of its silencing (knock down), and to α-tubulin (TUB), used as a loading control, are also shown.

**Fig. 2:**
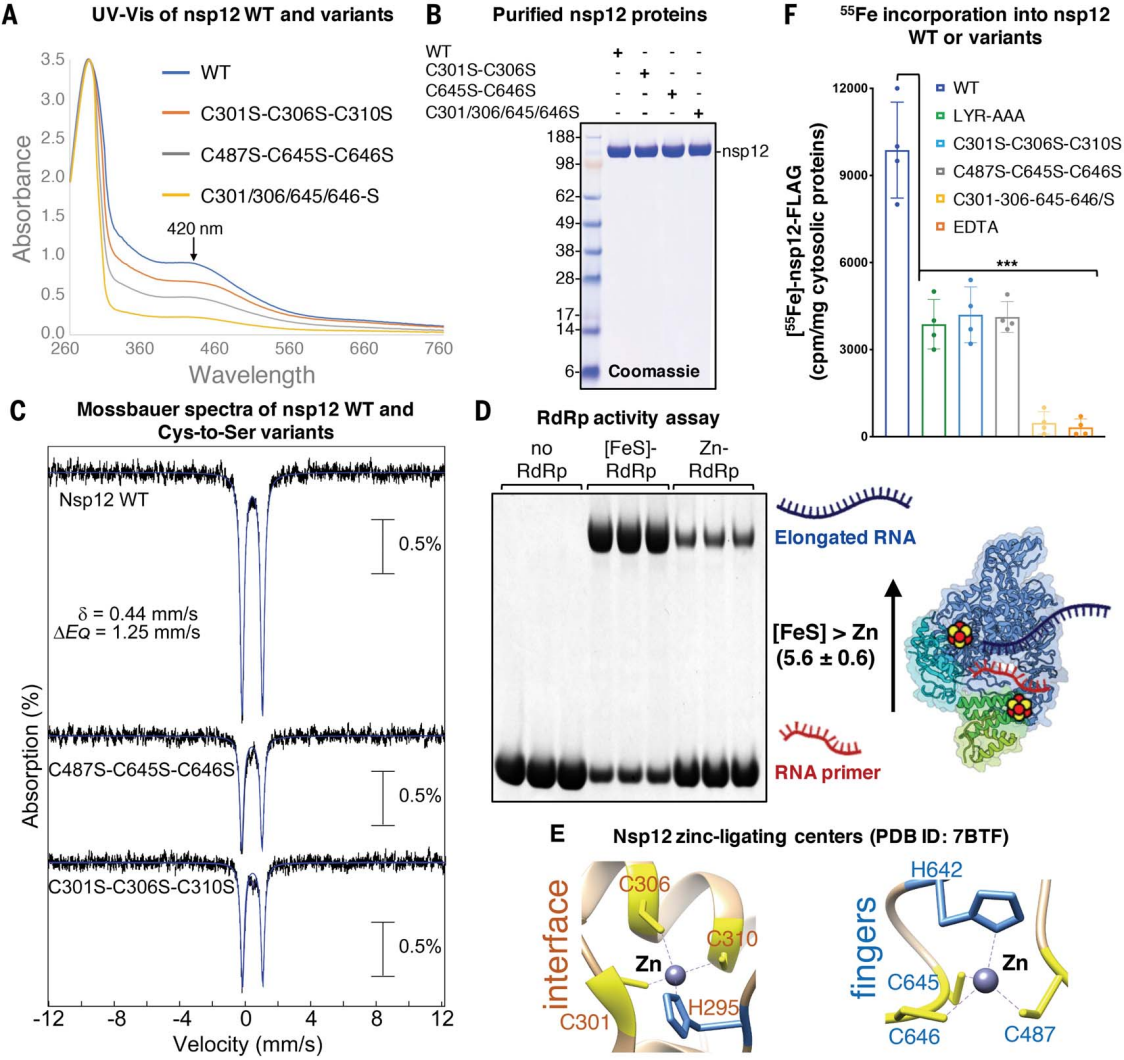
Evidence for ligation of two Fe-S metal cofactors by nsp12. (**A**) UV-vis spectra of nsp12 WT or variants of the cysteine residues in the two metal ligating centers. (**B**) Representative Coomassie blue staining of purified nsp12 WT or variants analyzed in (A). (**C**) Mössbauer spectra of nsp12 WT and variants exhibited the parameters typical of [Fe_4_S_4_] clusters. For each of the two nsp12 Cys-to-Ser variants, ~95% of iron was still associated with a quadrupole doublet that matched parameters of WT nsp12. (**D**) RNA polymerase activity of anoxically purified RdRp ([Fe-S]-RdRp at 1 μM) and aerobically purified RdRp reconstituted with zinc and containing two zinc ions per protomer (Zn-RdRp at 1 μM) (*n* = 4). (**E**) Conserved zinc-binding motifs in SARS-CoV-2 nsp12 [Protein Data Bank (PDB) ID 7BTF] (*8*) rendered in the ribbon representation. H295-C301-C306-C310 ligate zinc at the interface between the NiRAN and the catalytic domain, whereas the C487-H642-C645-C646 residues ligate zinc in the catalytic domain. (**F**) Levels of radiolabeled iron (^55^Fe) incorporated into nsp12 WT or variants, as indicated. ^55^Fe content of nsp12 treated with the chelator EDTA is also reported to provide a control for the complete loss of ^55^Fe in the protein (*n* = 4). Significance was determined by two-way ANOVA and Sidak’s multiple comparisons test. Mean ± 95% CI. ***P < 0.001.

Having recently demonstrated that we are able to predict the presence of Fe-S cofactors in candidate proteins based on the identification of specific amino acid sequence motifs (*27*), we analyzed the primary sequences of SARS-CoV-2 proteins to investigate whether any might incorporate Fe-S clusters. We identified two highly conserved LYR (leucine-arginine-tyrosine)–like motifs (fig. S2A) in nsp12 that have been previously characterized as potential binding sites for the cochaperone HSC20 (also known as HSCB) of the Fe-S biogenesis machinery (*27*–*30*), which facilitates Fe-S cluster transfer from the main scaffold protein, ISCU (iron-sulfur cluster assembly scaffold), to recipient proteins (fig. S2B). To assess whether the LYR-like motifs were involved in direct binding of nsp12 to HSC20, we incubated full-length SARS-CoV-2 nsp12 wild type (WT) or variants wherein either or both LYR motifs were replaced by alanines (A) (fig. S2C) with purified HSC20. Nsp12 WT bound HSC20, indicating that the RdRp subunit interacts directly with the cochaperone (Fig. 1A). Substitution of either of the two LYR motifs with alanines decreased the amount of bound HSC20 (Fig. 1A), which was even more profoundly diminished by loss of both motifs in nsp12^VYR/LYR-AAA^ (Fig. 1A). Coimmunoprecipitation (co-IP) experiments in Vero E6 cells and mass spectrometry analysis confirmed that nsp12 transiently interacted with HSC20 and with components of the de novo Fe-S cluster (the chaperone HSPA9, the cysteine desulfurase NFS1, and the main scaffold ISCU) and cytoplasmic Fe-S (CIA) biogenesis (CIAO1, MMS19, and FAM96B) machineries (Fig. 1, B and C; fig. S2D; and data S1), suggesting that these interactions may be required for Fe-S cluster acquisition by nsp12. To investigate whether nsp12 coordinated an Fe-S cluster, we quantified ^55^Fe incorporation into the protein expressed in cells transfected with either a pool of nontargeting small interfering RNAs (NT siRNAs) or with siRNAs against the initial Fe-S biogenesis scaffold, ISCU. In control cells (NT siRNAs), nsp12 WT bound radiolabeled iron (8312 ± 775 cpm/mg of cytosolic proteins) (Fig. 1, D and E), whereas nsp12 that lacked the LYR motifs did not interact with HSC20 and bound significantly less iron (250 ± 92 cpm/mg of cytosolic proteins) (Fig. 1, D and E). Nsp12 expressed in cells silenced for ISCU (si-ISCU) failed to incorporate iron (Fig. 1, D and E). Taken together, these results demonstrate that nsp12 binds iron, likely in the form of an Fe-S cluster. Nsp12 expressed in Expi293F mammalian cells and purified anoxically exhibited a shoulder at ~420 nm in its ultraviolet–visible (UV-vis) absorption spectrum (Fig. 2, A and B, and fig. S3, A and B), suggesting that it harbored one or more Fe-S clusters (*31*, *32*). To determine the type and stoichiometry of the Fe-S cluster(s), a ^57^Fe-enriched nsp12-FLAG sample was analyzed by Mössbauer spectroscopy (Fig. 2C). The 4.2-K Mössbauer spectrum collected in a 53-mT magnetic field applied parallel to the direction of gamma radiation (Fig. 2C) shows the presence of a single quadrupole doublet with parameters typical of [Fe_4_S_4_]^2+^ clusters [isomer shift (δ) of 0.44 mm/s and quadrupole splitting parameter (Δ*E*_Q_) of 1.25 mm/s, blue line] (*33*). Wild-type nsp12 bound 7.5 ± 0.35 iron atoms per monomer, and we thus interpret the Mössbauer spectrum as two [Fe_4_-S_4_]^2+^ clusters. The X-band electron paramagnetic resonance (EPR) spectrum, recorded at 20 K, showed no signal (fig. S3C), ruling out the presence of Fe-S clusters with a half-integer spin ground state. However, upon reduction with dithionite, EPR signal characteristics of [Fe_4_S_4_]^+^ clusters were observed (fig. S3D) (*34*). Notably, the nsp12-nsp7-nsp8 complex anoxically purified with the Fe-S cluster(s) showed markedly increased binding to the template and RNA primer (fig. S4) and increased polymerase activity relative to the aerobically purified complex that contained two zinc ions per protomer (Fig. 2D and fig. S4).

The available cryo-EM structures of the RdRp complex have assigned two chelated zinc ions in the highly conserved metal binding motifs of nsp12 composed of H295-C301-C306-C310 at the interface between the NiRAN (nidovirus RdRp-associated nucleotidyltransferase) domain and the catalytic domain and of C487-H642-C645-C646 in the fingers of the catalytic domain (*7*–*11*) (Fig. 2E and fig. S1; see supplementary text). By replacing selected cysteines with serines and characterizing the variant nsp12 proteins, we tested the hypothesis that the two [Fe_4_-S_4_] clusters are coordinated by these motifs. The two variants lacking any one of the set of three Cys residues of either the interfacial motif (nsp12^C301S-C306S-C310S^) or the catalytic domain (nsp12^C487S-C645S-C646S^) (replaced by Ser) contained 3.8 ± 0.2 and 3.67 ± 0.3 Fe per nsp12 protomer, respectively, and exhibited approximately half of the absorbance at 420 nm (Fig. 2, A and B, and fig. S3, A and B) and half of the ^55^Fe radiolabel seen for the WT nsp12 (Fig. 2F). The 4.2-K/53-mT Mössbauer spectra of these two variants revealed that ~95% of Fe is associated with the quadrupole doublet with the same parameters deduced from the spectrum of WT nsp12, thus revealing the presence of one [Fe_4_S_4_]^2+^ cluster in the unmodified binding site (Fig. 2C). The 20-K X-band EPR spectra of the variants after they were treated with sodium dithionite are also consistent with the presence of one [Fe_4_S_4_]^2+^ cluster (fig. S3D). A variant lacking a total of four cysteines from both motifs (nsp12^C301S-C306S-C645S-C646S^) did not bind Fe and had no absorbance at 420 nm, consistent with the notion that both [Fe_4_S_4_] cluster binding sites had been eliminated (Fig. 2, A and B, and fig. S3, A and B). The two [Fe_4_S_4_ ]^2+^ clusters incorporated in a mammalian overexpression system are thus ligated by cysteine residues located in the two zinc-binding sites identified in the cryo-EM structures.

**Fig. 3:**
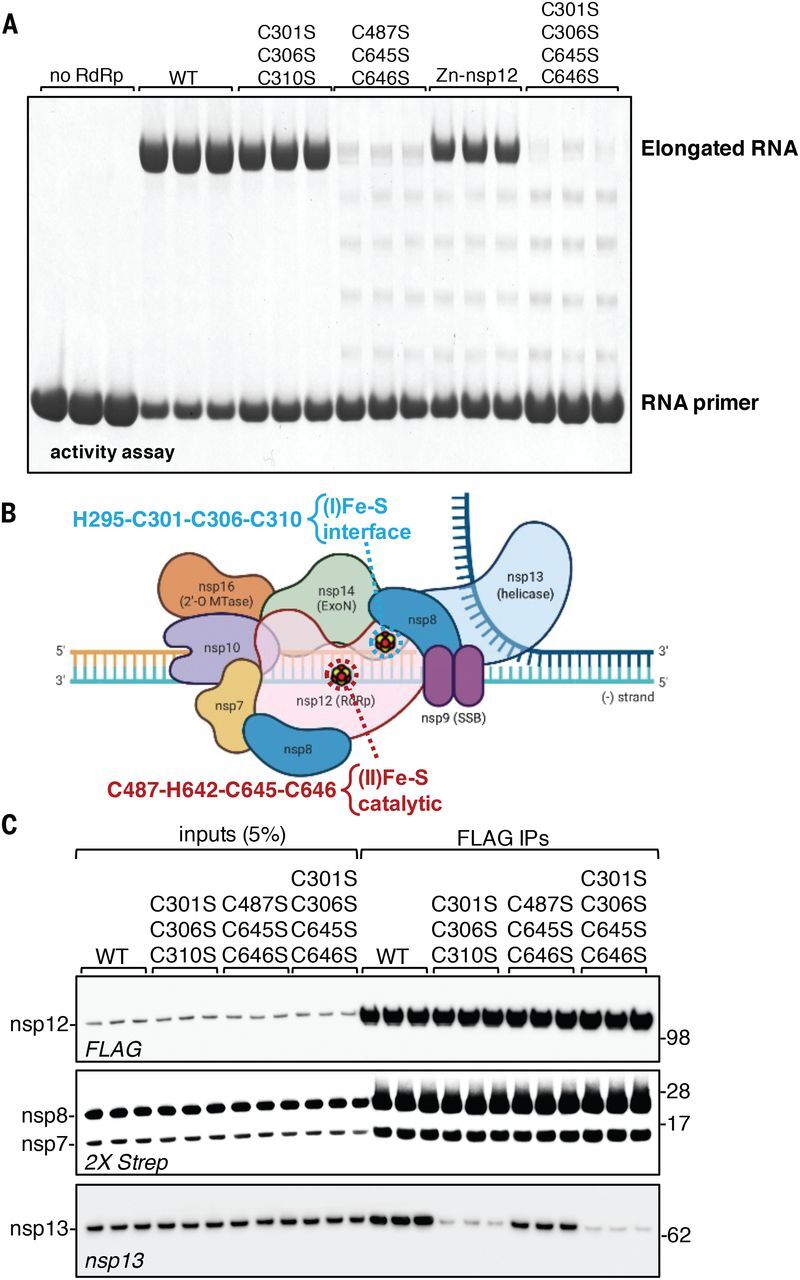
Fe-S cluster sites in nsp12 are important for activity and interactions with nsp13. (**A**) RNA polymerase activity of anoxically purified RdRp (all lanes except Zn-nsp12) (RdRp at 1 μM) and of aerobically purified and Zn-reconstituted RdRp containing two zinc ions per protomer (three technical replicates are shown; *n* = 4). (**B**) Schematic of the complex required for coronaviral replication (*10*), in which the two Fe-S clusters and their coordination spheres are highlighted. ExoN, exoribonuclease; SSB, single-stranded DNA-binding protein; 2′-O MTase, 2′-O methyltransferase. (**C**) Co-IP of nsp12 WT or variants recombinantly expressed in Vero E6 cells cotransfected with helicase nsp13 and accessory factors nsp7 and nsp8 (Strep II tagged) probed with antibodies against FLAG, Strep II, or nsp13 (three technical replicates are shown; *n* = 4).

We next aimed to characterize the role of the two Fe-S clusters in the RdRp. Functional studies revealed that the [Fe_4_S_4_] cluster in the catalytic domain of nsp12 is required for the RNA polymerase activity of the nsp12-nsp7-nsp8 complex (Fig. 3, A and B), in addition to presumably maintaining structure. In fact, the absence of the cysteine ligands in the catalytic domain in the nsp12^C487S-C645S-C646S^ variant caused a more profound decrease in the polymerase activity than was observed in the zinc complex (Fig. 3A), suggesting that Zn, by coordinating the same cysteine residues, can partially fulfill the structural role of the Fe-S cluster, preserve the architecture of the fingers subdomain, and maintain some polymerase activity, which is strictly associated with the palm of the catalytic domain (*8*, *11*). Fe-S enzymes involved in DNA and RNA metabolism have often been mischaracterized as zinc-containing proteins, as Fe-S clusters readily undergo oxidative degradation during standard aerobic purification procedures of proteins, allowing zinc to coordinate the same cysteine residues. Moreover, zinc-containing enzymes have been shown to retain activity in vitro on short templates (*14*, *35*), which previously supported the conclusion that zinc was the physiological cofactor of these enzymes. Fe-S clusters in nucleic acid metabolism enzymes have been thought to participate directly not in catalysis but in modulating binding of the enzyme to the template and/or to other components of the replication complex (*26*, *36*, *37*), as well as in increasing processivity and enabling repair through a proposed charge-transfer mechanism (*38*, *39*). Consistent with the notion that zinc is likely not the physiological cofactor in several viral replicases that have so far been crystallized with chelated zinc ions, supplementation with zinc has been reported to inhibit replication in several cell culture models of viral infection (*40*–*42*). Loss of the [Fe_4_-S_4_] cluster ligated by H295-C301-C306-C310, which is located at the interface between the NiRAN and the catalytic domain of nsp12, had minimal effect on the RNA polymerase activity (Fig. 3, A and B). However, loss of this cluster profoundly diminished the interaction with the helicase nsp13 (Fig. 3, B and C), which is an essential component of the replication complex.

**Fig. 4:**
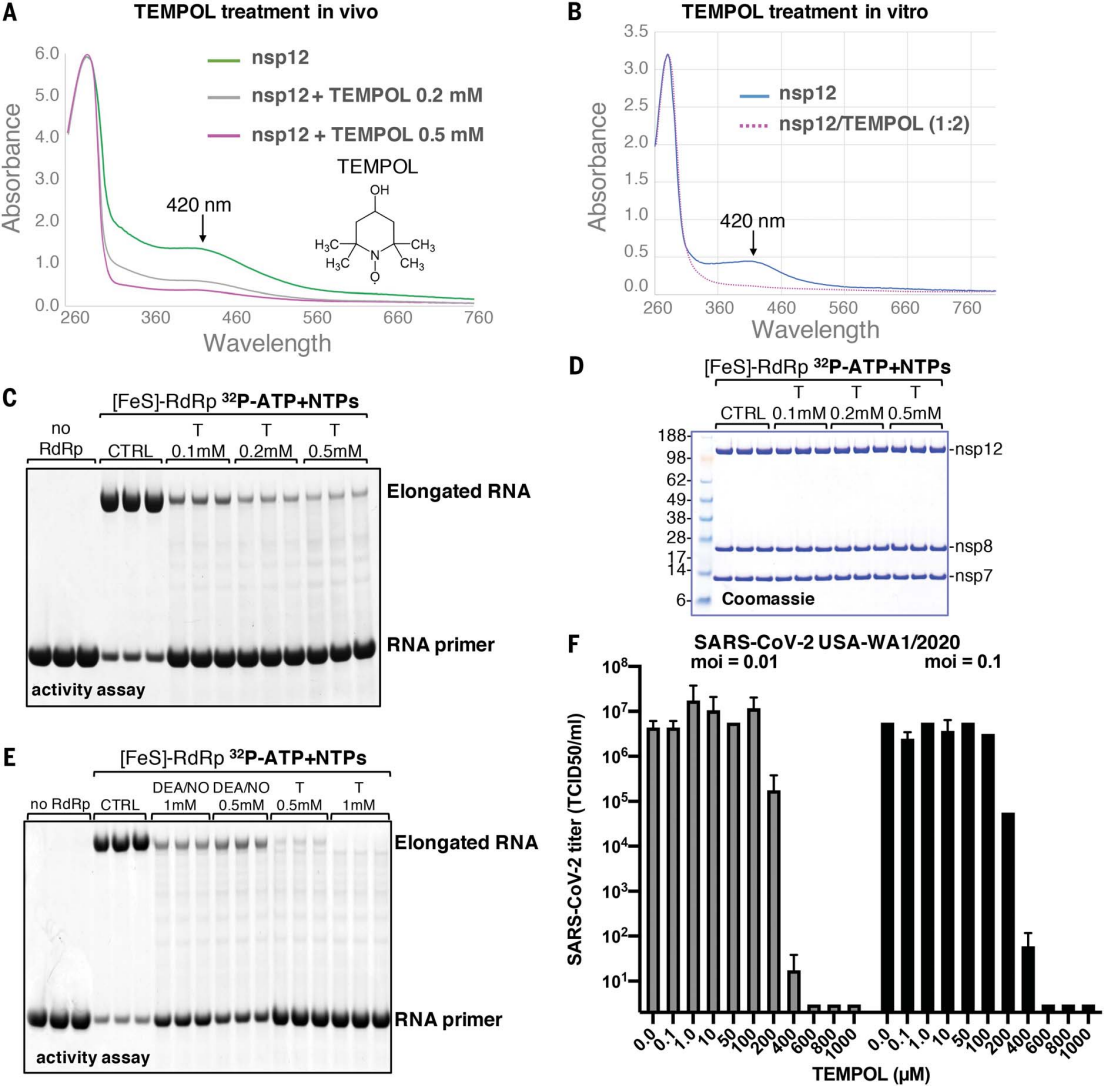
The stable nitroxide TEMPOL potently inhibited the RdRp by causing disassembly of its Fe-S clusters and blocked viral replication in cell culture models of SARS-CoV-2 infection. (**A**) UV-vis spectra of nsp12 anoxically purified from Expi293F control cells and from cells treated with TEMPOL. (**B**) UV-vis spectra of purified nsp12 and of purified nsp12 incubated with TEMPOL (1:2 ratio nsp12:TEMPOL) for 10 min. (**C**) RNA polymerase activity of the RdRp complexes anoxically purified from control and TEMPOL-treated (T) Expi293F cells. (**D**) Representative Coomassie staining of the RdRp complexes analyzed for activity in (C). (**E**) RNA polymerase assay of the RdRp complexes (at 1 μM) anoxically purified from control or DEA/NO- or TEMPOL-treated Vero E6 cells, as indicated (*n* = 4). (**F**) Titer of infectious virus produced at 48 hours measured by TCID_50_ (median tissue culture infectious dose) assay in Vero E6 cells infected with SARS-CoV-2 at a multiplicity of infection (moi) of 0.1 or 0.01 (*n* = 3).

We attempted to exploit the sensitivity of Fe-S clusters to oxidative degradation (*43*) to prevent coronavirus replication in cell culture models. Previous studies have shown that a stable nitroxide, TEMPOL (4-hydroxy-2,2,6,6-tetramethylpiperidin-1-oxyl), was beneficial in two different animal models of human conditions through its ability to oxidize and disassemble the Fe-S cluster of cytosolic aconitase (IRP1), thereby converting it into the iron responsive element (IRE)–binding apo-form (*44*–*46*). RdRp isolated from Expi293F cells that had been treated with TEMPOL (Fig. 4A) had diminished absorbance at 420 nm relative to the complex isolated from untreated cells, indicative of loss of the Fe-S clusters of nsp12. Likewise, treatment with TEMPOL of the Fe-S cluster–containing protein in vitro caused loss of absorbance in the same region (Fig. 4B). Either treatment resulted in loss of polymerase activity (Fig. 4, C to E). The TEMPOL treatment of cells did not impact the activities of several mitochondrial Fe-S enzymes, including the respiratory complexes and mitochondrial aconitase (ACO2), and the cytosolic Fe-S enzyme dihydropyrimidine dehydrogenase (DPYD) (figs. S5 and S6, A to F), nor did it cause any cytotoxicity at doses up to 5 mM (fig. S6G). TEMPOL treatment also did not affect the interactions of nsp12 with the components of the Fe-S and CIA biogenesis machinery from which nsp12 acquires its Fe-S clusters (fig. S7). We thus infer that TEMPOL directly reacts with Fe-S clusters in RdRp, leading to their degradation.

In support of this mechanism of action, diethylamine nonoate (DEA/NO), a nitric oxide donor that readily reacts with Fe-S clusters to form dinitrosyl complexes with diminished absorbance (*47*, *48*), also inhibited the RdRp (Fig. 4E and fig. S8), although less effectively than TEMPOL. We found that TEMPOL was both a more potent RdRp inhibitor (fig. S9) and synergized with remdesivir (RDV) (fig. S10), a nucleoside analog that has been used to target the replication of SARS-CoV-2 (*49*). RDV was notably less effective against the Fe-S–RdRp than the zinc-RdRp (fig. S11).

Having demonstrated a strong inhibitory effect of TEMPOL on the activity of the RdRp of SARS-CoV-2, we asked whether TEMPOL might exhibit antiviral activity against live virus replication. Vero E6 cells were infected with the SARS-CoV-2 USA-WA1/2020 isolate in the presence of increasing concentrations of TEMPOL (range: 0.1 to 1 mM). TEMPOL exhibited strong antiviral activity at concentrations above 0.2 mM. Viral titers were reduced by more than 5 log_10_ in the presence of 0.4 mM TEMPOL, which is reported to have a 50% cytotoxic concentration (CC50) greater than 100 mM (*50*). Our studies present a molecular basis for pursuing TEMPOL—with its low cytotoxicity and known access to tissues relevant for COVID-19 infection (*51*, *52*)—and other related stable nitroxides as potential SARS-CoV-2 therapies during active viral infection.

## Supplementary Materials

science.sciencemag.org/content/373/6551/236/suppl/DC1
Materials and Methods Supplementary Text Figs. S1 to S11 References (*54*–*66*) MDAR Reproducibility Checklist Data S1

## Appendix

View/request a protocol for this paper from *Bio-protocol*.
